# Contrasting Evolutionary Dynamics of the Developmental Regulator PAX9, among Bats, with Evidence for a Novel Post-Transcriptional Regulatory Mechanism

**DOI:** 10.1371/journal.pone.0057649

**Published:** 2013-02-28

**Authors:** Caleb D. Phillips, Boyd Butler, John W. Fondon, Hugo Mantilla-Meluk, Robert J. Baker

**Affiliations:** 1 Department of Biological Sciences, Texas Tech University, Lubbock, Texas, United States of America; 2 Department of Biology, University of Texas at Arlington, Arlington, Texas, United States of America; Laboratoire de Biologie du Développement de Villefranche-sur-Mer, France

## Abstract

Morphological evolution can be the result of natural selection favoring modification of developmental signaling pathways. However, little is known about the genetic basis of such phenotypic diversity. Understanding these mechanisms is difficult for numerous reasons, yet studies in model organisms often provide clues about the major developmental pathways involved. The paired-domain gene, PAX9, is known to be a key regulator of development, particularly of the face and teeth. In this study, using a comparative genetics approach, we investigate PAX9 molecular evolution among mammals, focusing on craniofacially diversified (Phyllostomidae) and conserved (Vespertilionidae) bat families, and extend our comparison to other orders of mammal. Open-reading frame analysis disclosed signatures of selection, in which a small percentage of residues vary, and lineages acquire different combinations of variation through recurrent substitution and lineage specific changes. A few instances of convergence for specific residues were observed between morphologically convergent bat lineages. Bioinformatic analysis for unknown PAX9 regulatory motifs indicated a novel post-transcriptional regulatory mechanism involving a Musashi protein. This regulation was assessed through fluorescent reporter assays and gene knockdowns. Results are compatible with the hypothesis that the number of Musashi binding-elements in PAX9 mRNA proportionally regulates protein translation rate. Although a connection between morphology and binding element frequency was not apparent, results indicate this regulation would vary among craniofacially divergent bat species, but be static among conserved species. Under this model, Musashi’s regulatory control of alternative human PAX9 isoforms would also vary. The presence of Musashi-binding elements within PAX9 of all mammals examined, chicken, zebrafish, and the fly homolog of PAX9, indicates this regulatory mechanism is ancient, originating basal to much of the animal phylogeny.

## Introduction

The genetic mechanisms underlying human development as well as the morphological variation observed in natural populations remain poorly understood. Yet, it is clear that proper development is the result of complex regulation of developmental signaling pathways. This has been partly manifested in the anomalous human phenotypes often resulting from single, or a few, mutational events [1]. Furthermore, experiments using model organisms have shown that a subset of genes of regulatory function have major effect on craniofacial and dental development [2]. Although such findings derived from model organisms are highly relevant, identifying salient genomic changes underlying morphological adaptation is complicated by the general inability to produce test crosses, the difficulty of separating neutral and functional genetic variation, and understanding the relative importance of focusing on regulatory versus open-reading frame variation. In light of this, the study of interspecific morphological differences often relies on candidate genes identified in model systems. The benefit of candidate gene studies is that they exploit *a priori* established links between genotype and phenotype to enable the characterization of evolutionarily significant molecular divergences in genetically intractable non-model organisms.

The paired-box gene family of proteins (PAX) is an evolutionarily conserved and ancient gene family, having originated during the pre-Cambrian [3]–[4]. One member of this family, PAX9, is considered a candidate gene for craniofacial and dental evolution. PAX9 is known to play an important role in development as both a transcription factor and through protein-protein interactions [5]–[8]. PAX9 expression is necessary for proper development of the craniofacial and dental regions, and is also expressed during development of the vertebral column, tongue, limbs, and other organs [9]–[13]. Mutations in coding and putative regulatory regions of PAX9 are often associated with phenotypic abnormalities. Human phenotypes associated with PAX9 mutations are most commonly various forms of tooth agenesis [14]–[18]. PAX9 knock-outs are non-viable owing to defective development of the pharyngeal pouches resulting in malformed palatal and neck structures, and also exhibit tooth agenesis and polydactyly [9]. Clearly, improving models for PAX9 sequence evolution and how PAX9 is regulated are needed to better understand complex PAX9 involved developmental signaling pathways [9]–[10]. For example, it is reasonable that expression levels of Pax9 during the developmental process influence downstream effector expression and hence, the timing of cellular differentiation. Variance in this process would have implications to both the evolution of phenotypes in nature, as well as candidate mechanisms for anomalous human phenotypes.

**Figure 1:**
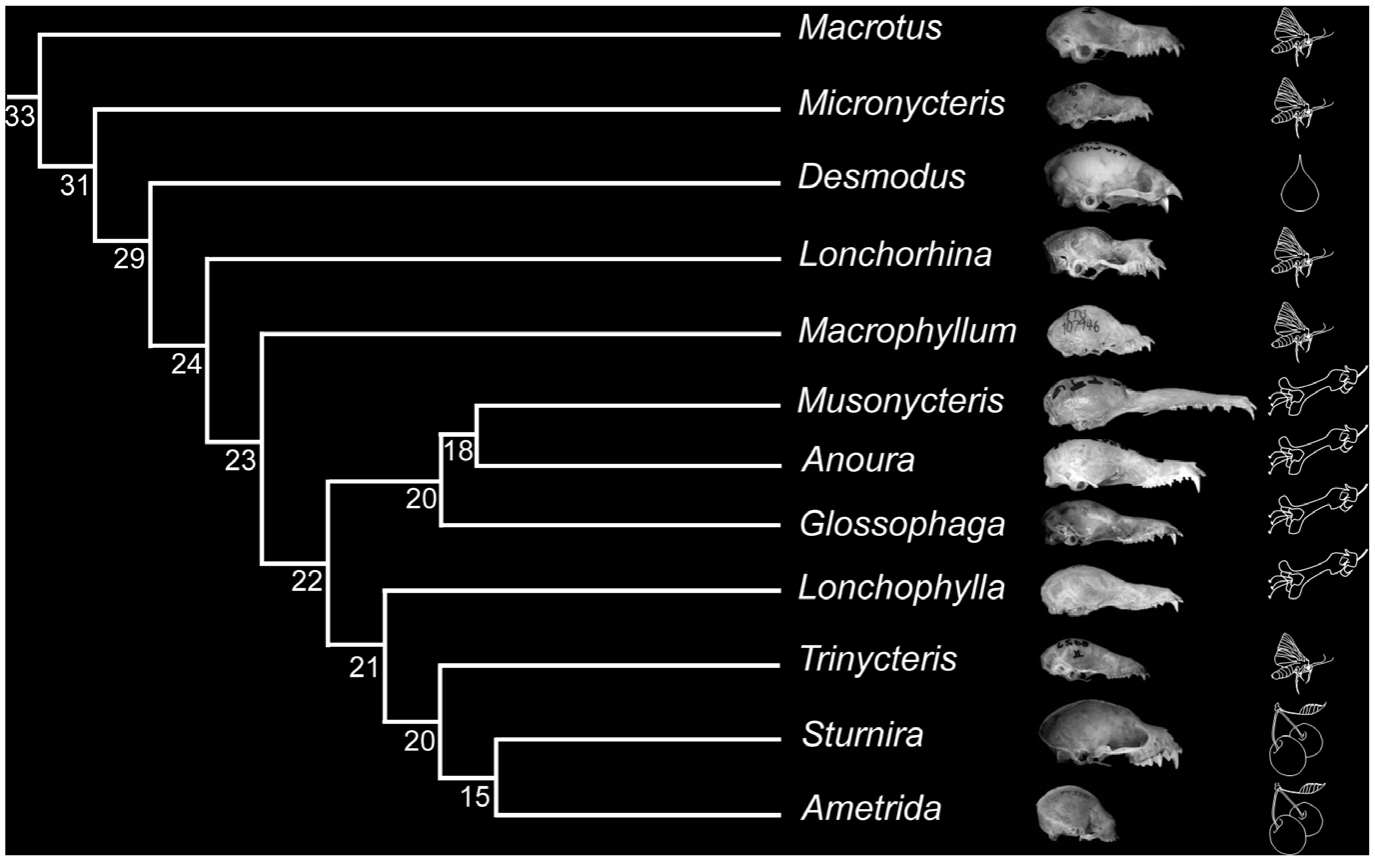
Cladogram reconstructed from [22], [28]–[29] for phyllostomid bats included in this study. Node values are estimated divergence times taken from [22], [28]–[29]. Each leaf of the cladogram includes genus, lateral image of skulls, and symbols of insect, blood, flower, or fruit to indicate dietary strategy of that genus.

Highly variable intra-ordinal and intra-familial patterns of craniofacial and dental evolution are observed within the order Chiroptera. Currently, 19 families of bats are recognized [19]–[20]. Although the family Phyllostomidae is thought to be among the youngest families, this group displays the most craniofacial, dental, and dietary diversity [21]–[22]. It is hypothesized that the morphological variability observed within Phyllostomidae is the result of niche adaptation as multiple dietary strategies are represented in the family, including insectivory, frugivory, nectarivory, sanguivory, omnivory, and carnivory [21]–[22]. Comparison of lineages illustrates often dramatic craniofacial morphological differences (Figure 1) for structures in which Pax9 is expressed during development. For example, there is extensive variation in rostral length, cranial morphology, dental formula, tooth shape, as well as extreme variation in tongue length among nectarivorous lineages. By comparison, the family Vespertilionidae, which is perhaps only slightly older than the Phyllostomidae [19], is very speciose, mostly represented by insectivorous taxa, and all maintain similarly conserved craniofacial and dental morphologies. The difference in occurrence of morphological diversification observed across these families enabled the development of a case-control type experimental design to understand open-reading frame and regulatory sequence evolution of PAX9. We investigated evolutionary rate, substitution patterns, and convergence of PAX9 in phyllostomids, vespertilionids, and other mammalian orders to understand if patterns were compatible with the hypothesis that PAX9 open-reading frame evolution has contributed to morphological diversification. Through comparison of regulatory regions, we developed a hypothesis for a novel translational regulatory mechanism, for which we provided experimental validation, and discussed potential developmental and evolutionary ramifications.

## Materials and Methods

### Sampling and Sequence Collection

Approximately 50 milligrams of tissue (heart, kidney, or liver) from 12 phyllostomids, 13 vespertilionids, one miniopterid, and two pteropodid species were loaned from the Genetic Resources Collection of the Natural Science Research Laboratory at Texas Tech University. Sampling included representatives of each of the major morphological diversifications associated with dietary diversifications within family Phyllostomidae (morphologies characteristic of insectivorous, sanguivorous, frugivorous, and nectarivorous lineages) as well as species of family Vespertilionidae (and one Miniopteridae, a sister family) selected to include a similar amount of evolutionary time as that sampled in the phyllostomids. Deeper evolutionary comparisons were also made to another divergent family, the family Pteropodidae (Old World megabats), as well as other mammalian orders and super-orders (outlined below). The two pteropodid species included are nectarivorous and exhibit some of the cranial and dental morphologies characteristic of this life history (elongated rostrum, increased spacing between molars, and reduced molar morphological diversity) that are shared with the four genera of nectarivorous phyllostomids also examined in this study (although derived from independent insectivorous ancestors). Museum identifications, taxonomic and dietary classifications are available in Table S1.

Genomic DNAs were isolated by phenol/chloroform extraction and precipitated isolations were rehydrated in TE buffer to a concentration of 125 ng/µl, determined by agarose electrophoresis and spectrophotometry. Two-hundred fifty ng of template genomic DNA was used in 50 µl PCR reaction volumes with 12.5 µmoles each deoxynucleoside, 1 µl Phire polymerase (New England Biolabs, Ipswich, MA), manufacturer buffer, and 25 pmoles each primer (all primers are described in Table S2 and Table S3). Amplicons were excised from 0.8% agarose gels and purified using silica membrane columns (Qiagen, Valencia, CA). Between 10 and 150 ng of amplicon was dehydrated depending on fragment size and cycle sequenced with 15 pmoles sequencing primer using Applied Biosystems BigDye Terminator Cycle Sequencing Kit version 3.1 (Applied Biosystems Inc., Foster City, CA). Sequencing products were purified by centrifugation through sephadex columns (Princeton Separations, New Jersey, NJ), dehydrated, and resuspended in formamide. Sequencing was performed on an ABI 3100-Avant Genetic Analyzer. Sequences were aligned in Sequencher version 4.9 (Gene Codes Corporation, Ann Arbor, MI) and manually edited.

Additional sequences for PAX9 were retrieved from GenBank to provide broader taxonomic comparison. The order Primates was represented by 15 species using data previously reported by [23] (GenBank accession numbers DQ067505–DQ067556). Additional PAX9 sequences were retrieved from genome sequence data from *Myotis lucifugus*, *Canis familiaris*, *Felis catus*, *Bos taurus*, *Mus musculus*, *Rattus norvegicus*, *Loxodonta africana*, and *Homo sapiens*. These sequences enabled a comparison across basal nodes of the Eutherian phylogeny. In addition, PAX9 UTR sequences were also retrieved for *Gallus gallus* and *Danio rerio*. Sequences for these bird and fish species were included to provide a general understanding about the timing of origin of identified putative regulatory motifs.

### Open-reading Frame Sequence Evolution

Multiple tests and summary statistics were calculated to describe PAX9 evolutionary patterns (Table 1). Best fit models of molecular evolution for the nucleotide and predicted amino acid data were determined using the Bayesian Information Criterion and using neighbor-joining trees generated from the alignment. A Maximum Likelihood test of the molecular clock was performed to test if mutational distances from the root of the tree to the tips were equidistant. To quantify the overall pattern of selection a codon-based test for strict neutrality by averaging across sequence-pairs was performed with variance of the difference obtained through 1000 bootstrap replicates [24]. Codon-by-codon estimated dN-dS ratios (based on joint Maximum Likelihood reconstructions of ancestral states) were calculated to identify codons exhibiting an excess of non-synonymous substitutions (P values reflect the probability of rejecting the null hypothesis of neutral evolution and are the exact binomial probability of obtaining the observed or more biased numbers of synonymous and nonsynonymous changes for each codon site [25]). These analyses were performed using MEGA 5 [26] and codon-by-codon selection tests were performed using HyPhy [27] within MEGA 5.

**Table 1:** Major analyses implemented in this study with short descriptions for the information obtained from each analysis.

Analysis	Information
**Open-Reading Frame**	
Molecular clock test	Detects substitution rate heterogeneity across phylogeny
Neutrality tests	Detection of selection
Saturation plots	Characterizes recurrent substitution over time
Ancestral state reconstruction	Estimates mutational pathways along given phylogeny
Structural prediction	Deduces polypeptide secondary structure
Binomial probability	Random probability of observed substitution frequencies by exon
Reticulation network	Phylogenetic inference, uncertainty displayed as reticulations
**Untranslated Regions**	
Phylogenetic shadowing	Defines statistically quantifiable conserved motifs
Regulatory element scanning	Identifies presence of any known regulatory motifs
Reporter expression assay	Quantification of translational regulatory control
RNAi	Gene knockdown to implicate target in proposed function

Analyses are grouped into open-reading frame or regulatory subheadings, indicating to which major hypothesis each analysis was relevant.

Saturation plots were calculated to provide perspective about how synonymous and nonsynonymous mutations have accumulated among lineages at different evolutionary timescales. Measures of pairwise number of nucleotide and predicted amino acid differences were made among species of bat within families, and among species of primates. In addition, calculations of average pairwise distance were also made between orders. The orders represented by the above described sampling included Primates and Rodentia (super-order Euarchontoglires), Chiroptera, Carnivora, and Artiodactyla (super-order Laurasiaplacentalia), as well as Proboscidea (super-order Afrotheria). These measures of distance were regressed against time to most recent common ancestor (*t_mrca_*). Divergence times for the data set were synthesized from several studies including [22], [28]–[29] for the family Phyllostomidae, [20], [30] for the families Vespertilionidae and Miniopteridae, [31]–[35] for divergences within order Primates, and [36] for divergence times among mammalian orders.

Following the established evolutionary relationships among taxa, ancestral character states of the predicted amino acid variation were reconstructed following the rules of parsimony implemented in the Mesquite software package [37]. Variable positions and the frequency of recurrent substitution were plotted along a secondary structural prediction of PAX9 calculated using PSIPRED [38]. The location of the paired-binding domain reported by [7] was referenced here for additional structural relation of the distribution of mutations observed across the class Mammalia. Using the information on total detectable substitutions, cumulative binomial probabilities for the observed number of substitutions per exon were also calculated (the random probability of obtaining the same or greater number of amino acid replacements by chance, given the total number of replacements observed and the length of the exon). To examine the extent to which patterns of non-synonymous sequence evolution have resulted in phylogenetic convergence among lineages a bootstrapped neighbor-joining reticulation network based on uncorrected distances was constructed using SplitsTree4 version 4.12.3 [39]. This method of phylogenetic reconstruction was chosen because analysis provides information about the uncertainty of evolutionary relationships depicted as reticulations. Visualized reticulations (closed-loops in an otherwise bifurcating phylogenetic reconstruction) can provide greater clarification about sequence evolution among taxa than does nodal support values generally associated with bifurcating phylogenies. For comparison however, bootstrapped (1000 replicates) neighbor-joining trees were also estimated from nucleotide and predicted amino acid alignments incorporating the best-fit models of molecular evolution (determined above).

### Regulatory Motif Analyses and Reporter Assays

The 5′ and 3′ UTR of PAX9 from all bat species, *Homo sapiens*, *Canis familiaris*, and *Mus musculus* (genome reads with full coverage of the UTRs) were screened for the presence of conserved islands by phylogenetic shadowing. Conserved islands are regions of nucleotide conservation that are identified by sequence comparison among taxa and among nucleotide positions within an alignment. Identifying conserved islands is important because they disclose nucleotide regions that may serve regulatory functions. For this analysis the hidden Markov model (HMM) approach available in the eShadow program was implemented [40]. The analysis consisted of the construction of a multiple sequence alignment, describing the alignment as a percent variation plot, and statistical evaluation to detect conserved islands [40]. For the statistical analysis, open-reading frames were defined for rate calibration to train the Hidden Markov Model parameters used to signify conserved islands. Following conserved island identification, nucleotide sequences of both open-reading frames and UTRs were surveyed for the presence of known regulatory elements. Regulatory elements observed in mRNAs are often sites of mRNA-protein interactions and can function to promote regulation of mRNAs at the post-transcriptional level. To determine the presence of any such elements within PAX9, sequences from each bat species was screened for 46 known UTR embedded regulatory motifs using UTRscan [41]. For this analysis complete PAX9 sequences from *Homo sapiens*, *Canis familiaris*, *Mus musculus*, *Danio rerio*, and *Gallus gallus* were included, as well as open-reading frame sequences from the available primates. The purpose of including these divergent species in addition to bat taxa was to describe the evolutionary conservation of identified regulatory elements contained within PAX9.

**Figure 2:**
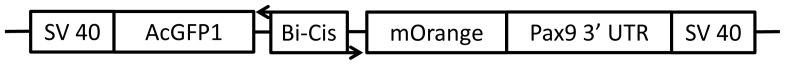
Diagram of reporter construct design. SV 40 = Simian virus 40 polyadenylation signal, AcGFP1 = green fluorescent protein, Bi-Cis = bicistronic promoter, mOrange = orange fluorescent protein, Pax9 3′ UTR = 3′ sequences of PAX9 from bat species described in the text. Experimental constructs differed only in the species from which PAX9 sequence was amplified. The control construct did not include a PAX9 3′ sequence.

The analyses of PAX9 disclosed the presence of Musashi binding-elements (MBEs) that varied in frequency among lineages (specific findings reported in Results). Musashi is involved in maintenance of stem cell state, cellular differentiation, and tumorigenesis and has been shown to accomplish this regulation through repression of target mRNAs [42]. To investigate the functionality of these identified regulatory motifs a fluorescent reporter assay was developed. A tetracycline responsive bicistronic expression vector (pTRE-Tight-Bi-AcGFP1; Clontech Laboratories, Mountain View, CA) was constructed to contain mOrange fluorescent protein followed by the 3′ 130 bp of the open-reading frame and the contiguous 3′ UTRs (Figure 2). The purpose of including the 3′ end of the open-reading frame as part of the heterologous 3′ UTR was to include the MBE identified in this region, as well as those found in 3′ UTRs. Multiple constructs were created that differed only in the specific bat lineage from which the PAX9 sequence was amplified. Bat lineages were selected to include the major phylogenetic and morphological divergence among nectarivorous taxa, the highly derived vampire bat lineage, and the observed frequency variation of Musashi binding elements among phyllostomids. Final vectors contained two MBEs (TK19556, *Musonycteris harrisoni*), two different spatial combinations of three MBEs (TK101009, *Desmodus rotundus*; TK104582, *Lonchophylla concava*), four MBEs (TK101008, *Glossophaga soricina*), or only the vector’s native simian virus 40 (SV40) polyadenylation signal containing no MBE, resulting in five unique expression constructs. In all constructs acGFP1 served as an internal control, being expressed in the opposite direction of the bicistronic promoter region from mOrange. Vectors were transfected (Nucleofector Reagent R; Lonza, Basel, CH) into Tet-Off Advanced HeLa cells (Clontech Laboratories, Mountain View, CA). HeLa was selected as the cell line to be used for these experiments because Western blotting confirmed the expression of both Musashi-2 and Musashi-1 (data not shown). Forty-eight hours post-transfection, relative fluorescence of both acGFP1 and mOrange was measured using a SpectraMax Gemini XPS plate reader (Molecular Devices, Sunnyvale, CA). Transfection and measurement for each cell line was replicated four times and the ratio of acGFP1 to mOrange for each measurement was taken as the comparable measure of reporter protein expression across cell lines and transfections. Expression levels across cell lines were compared using a Kruskal-Wallis test after a Shapiro-Wilk test indicated the data were not normally distributed (W = 0.9, P<0.01).

For each cell line transient Musashi knockdowns were created using RNAi. These experiments were carried out to confirm the role that Musashi played in any observed differences among lines during the experiments described above. Sets of three siRNAs for both Musashi-1 and Musashi-2 (Origene, Rockville, MD) were used at a concentration of 3 nM to silence Musashi expression. Knockdown curves indicated 95% silencing at 48 hours post-knockdown and this time point was used for subsequent measurements. Each cell line was transfected with siRNAs using lipofectamine 2000 (Invitrogen, Grand Island, NY) and fluorescence was measured as described above. The knockdown experiment was replicated three times for each cell line.

## Results

### Open-reading Frame Sequence Evolution

The open-reading frame of chiropteran PAX9 was found to be 1026 bp. Sequence coverage among bats was represented by only 1.1% missing data, and exons were sequenced in forward and reverse directions. Sequences are available from GenBank under accession numbers [KC549918–KC549944]. In order to characterize patterns of PAX9 open-reading frame variation several molecular analyses were conducted (Table 1). Using sequences of bats species generated for this study and others retrieved from GenBank (including several mammalian orders and primate taxa, see Materials and Methods) the best fit models of molecular evolution at the nucleotide and amino levels were T92+ G (0.9)+I (0.6) and JTT+G (0.05), respectively. The molecular clock was rejected at the nucleotide (P = 0.0001) and predicted amino levels (P = 0.0001). A history of purifying selection was signified through the overall test of strict neutrality (d_N_−d_S_ = −16.99, P = 0.0001). Similarly, codon-by-codon analysis disclosed no codon positions that were significant for positive selection (i.e., no positive d_N_-d_S_ were observed).

**Figure 3:**
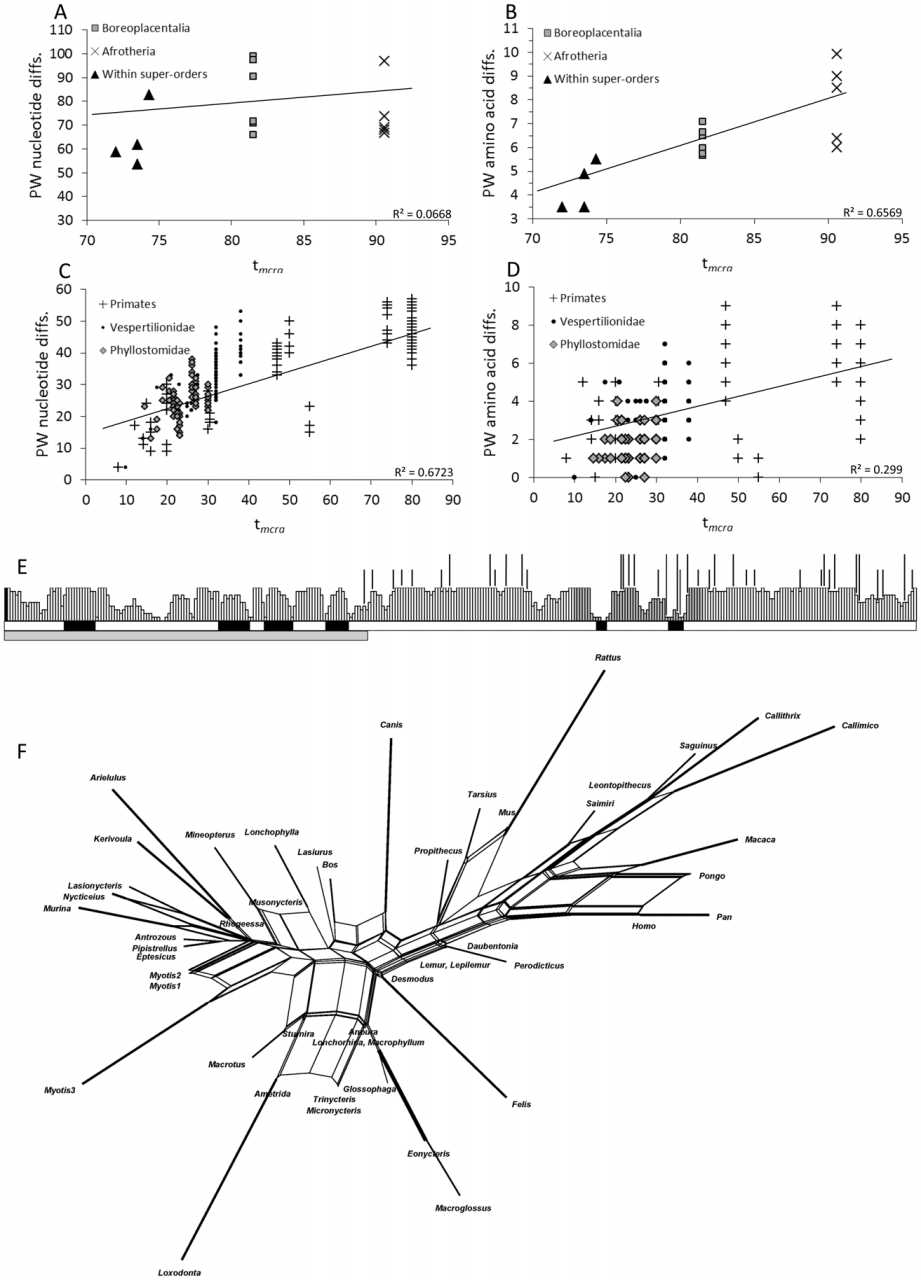
Patterns of PAX9 open-reading frame evolution. Although under tight purifying selection, PAX9 exhibits patterns of saturation and recurrent substitution contingent on the level of comparison (amino acid versus nucleotide) as well as the amount of evolutionary time considered in the data. A) and B) display the pairwise nucleotide and predicted amino acid differences among orders regressed against *t_mrca_*, respectively. C) and D) show similar plots, but among species from the families Phyllostomidae, Vespertilionidae and Miniopteridae, and the order Primates. E) Confidence in PSIPRED secondary structure prediction (greater confidence represented by larger bars) for each of the 341 residues of Pax9, and shading demarks exons. Directly below this histogram is the structural prediction in which white bars represent coiled structures and black represent helical regions. The solid grey bar at the bottom defines the limits of the paired-binding domain described by [7]. Vertical lines pointing to each codon position in the histogram indicate positions that vary across the mammalian taxa examined, and taller lines demark sights inferred to have accumulated recurrent substitutions. The number of inferred substitutions at these sights from left to right are as follows: 3, 2, 3, 2, 7, 4, 3, 4, 8, 6, 3, 2, 4, 4, 2, 3, 2, and 5. F) Reticulation network based on predicted amino acid translations. Closed loops in the network indicate homoplasies, and edge thickness is in proportion to bootstrap support.

In order to characterize the distribution genetic variation over evolutionary time, pairwise nucleotide and predicted amino acids differences at different taxonomic levels were regressed against *t_mrca_* (Figure 3a–d). The regression plots indicated saturation at the nucleotide level among orders (manifested as a weak linear relationship between pairwise differences and divergence time), but not within comparisons among bat families and among primates. Conversely, amino acid evolution was not heavily saturated in comparisons between orders, or through comparisons within bats and within primates.

Subsequent analyses were directed toward understanding the distribution of substitution variation and recurrent substitution across different polypeptide domains and exons. The plot of amino acid changes along the secondary structural prediction (Figure 3e) confirmed previous studies [23] indicating conservation of the paired-binding domain, although a single species of primate, *Callithrix jacchus*, has acquired a single change (Ala135Thr) one residue from the end of the paired-binding domain following the length reported by [7]. The octapeptide motif (residues 196–203; with a putative role in transcriptional regulation) was also found to be entirely conserved across mammals examined. However, parsimony reconstructions of ancestral character states described a history of recurrent substitution in areas downstream of the paired-binding domain and outside of the octapeptide motif. Among 38 variable positions, 18 were inferred to have undergone multiple substitutions among mammals, with a total of 87 substitutions inferred to have occurred over these 38 sites (an average of 2.3 changes per variable site). The specific number of changes inferred per site is included in the caption of Figure 3. For exons 2, 3, and 4 the cumulative probability for the observed number of nonsynonymous mutations given each exons length was found to be 0.61, 1.2×10^−11^, and 0.02, respectively.

Phylogenetic analysis by neighbor-joining reticulation network of the predicted amino acid translations (Figure 3f) illustrated recurrent substitution identified in the above analyses occurred both among orders and among families. The centrality of the network consisted entirely of reticulations. Similarly, low nodal support was observed for the predicted polypeptide neighbor-joining phylogeny (Figure S1). Generally, the primates, vespertilionids, and phyllostomids clustered together in separate parts of the network, although relationships within these taxonomic groups were confounded by clade-specific recurrent substitution. Several instances of misplacement of specific lineages were also observed. For example, *Loxodonta* was placed within a clade of phyllostomid bats, and rodents were positioned amongst primates. Among misplacements within bats, independently derived nectar feeders clustered on two separate occasions. This occurred once when the nectarivorous pteropodids *Macroglossus* and *Eonycteris* were positioned on a clade sister to the nectarivorous phyllostomid *Glossophaga* (the result of Ala236Ser in the pteropodids and Ala236Thr in Glossophaga). The second misplacement of nectar feeders occurred in the grouping of *Musonycteris* and *Lonchophylla*, two independently derived phyllostomid nectarivorous genera. This placement was the result of convergence to Glu252Asp. Of additional interest within phyllostomids was the observation that the morphologically similar [22], yet evolutionarily divergent, insectivorous genera *Micronycteris* and *Trinycteris* were identical at the amino acid level.

### Cis-regulatory Evolution

**Figure 4:**
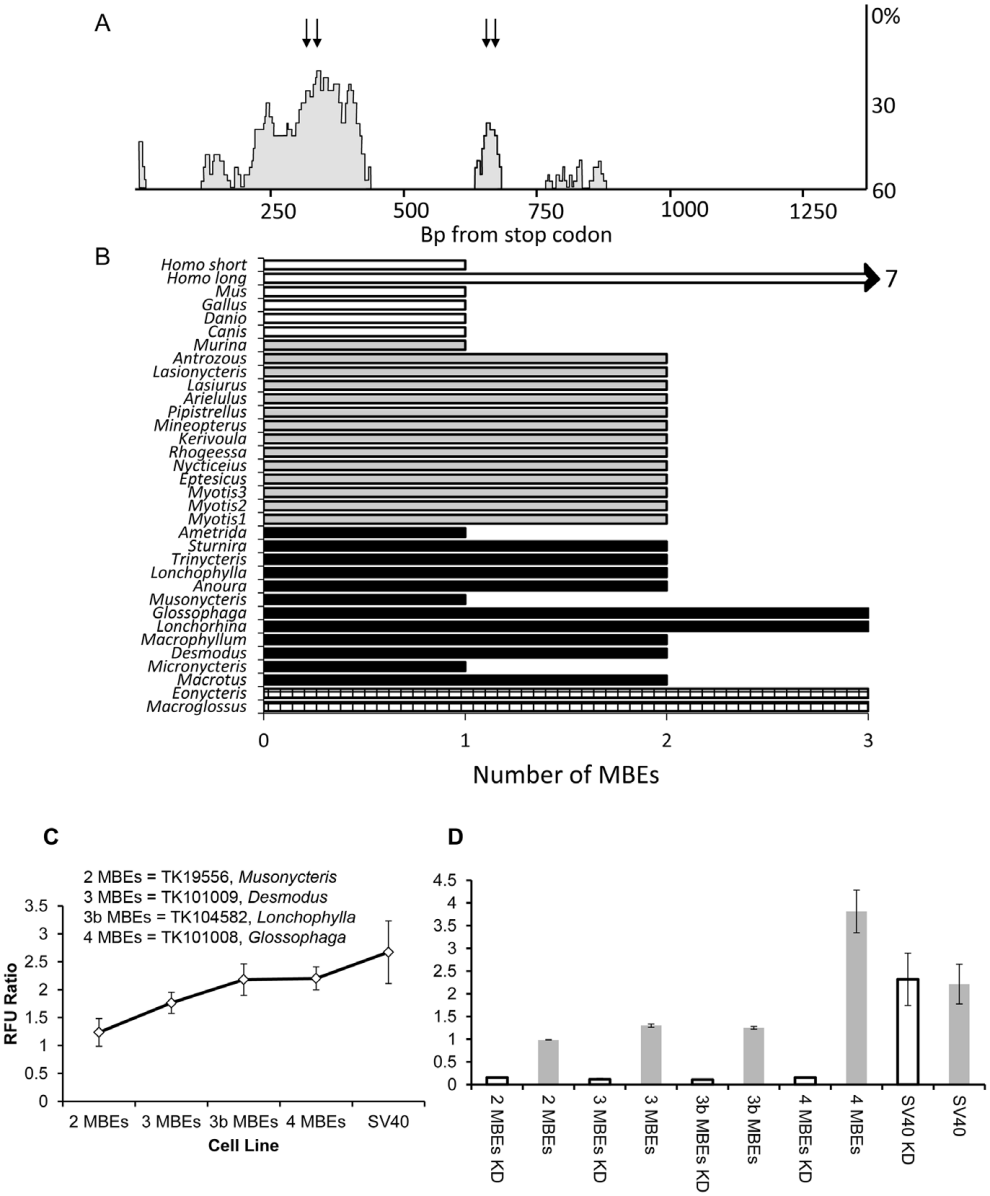
Comparative genetic and post-transcriptional reporter analysis. A) Patterns of nucleotide conservation across the 3′ UTR of PAX9 based on an alignment of *Homo*, *Canis*, *Mus*, and all bat species of this study, in which the vertical axis represents sequence divergence and the horizontal axis represents base pairs from the stop codon. Conserved domains are represented by grey areas and the locations of MBEs are demarked with arrows. The location of all seven MBEs occurring in a human alternative transcript are not show to conserve space (length >3Kb). B) Histogram summarizing the frequency of MBEs across taxa surveyed (checkered = pteropodids, black = phyllostomids, grey = vespertilionids, and white = non-bat taxa). *Homo* long and *Homo* short refer to the two alternative transcripts of PAX9 observed in humans. C) Results of the fluorescent reporter assay, with error bars representing standard mean error. Cell lines labeled as ‘3 MBEs’ and ‘3b MBEs’ are alternative spatial combination of this motif frequency and, SV40 represents the construct with only SV40 polyadenylation signal containing no MBE. D) Result of the knockdown assay. Cell lines denoted with KD indicate knockdown lines. Error bars represent standard mean error, and are not visible in some instances due to the error being within histogram bar thickness.

Outlier areas of nucleotide conservation within non-coding regions indicate potential regions of regulatory function. To identify any such motifs UTRs were analyzed for the presence of conserved islands. Both UTRs were found to contain conserved islands when comparisons included all non-bat mammalian taxa and phyllostomids or vespertilionids. When all samples were included the same conserved nucleotide domains were retained, although not statistically classifiable as conserved islands. Within the 5′ UTR a 146 bp conserved island 113 bp from the start codon was identified, and further analysis determined that this island is directly upstream from a putative internal ribosomal entry site. Within the 3′ UTR a 204 bp conserved island was identified 196 bp downstream of the stop codon. Next, nucleotide sequences of UTRs were surveyed for the presence of known regulatory elements identified in other studies using UTRscan [41]. This analysis revealed the presence of four Musashi-binding elements (MBEs; RT_n_AGT (n = 1 to 3)) within conserved regions of the 3′ UTR. However, among bats there was variation in the number of MBEs present in a given species 3′ UTR. Two MBEs were found to occur within the upstream conserved island, while another two were identified within a 64 bp region 588 bp downstream of the stop codon that is also highly conserved (Figure 4a). Among bats, there was variation in the number and combination of MBEs present in given species 3′ UTR; however this variation was almost exclusively observed in phyllostomids (Figure 4b). Within phyllostomids the four identified MBEs were observed in 92%, 0%, 25%, and 75% of species, respectively. Vespertilionids were fixed for presence of the first and fourth MBE, with the exception of *Murina*, which only had the first MBE. The first, second, and fourth MBEs were observed in both pteropodids examined. Analysis of the 3′ UTRs of human, dog, mouse, chicken, and zebrafish confirmed the presence of a single MBE within each of these species 3′ UTRs, with the exception of a proposed alternative transcript in human which contained seven MBEs. The discovery of MBEs within UTR regions led to an additional survey of open-reading frames for regulatory motifs. This analysis disclosed the presence of an additional MBE 32 bp from the stop codon in all chiropteran lineages except *Murina*.

These results, along with additional evidence gathered through literature and bioinformatic surveys (see Discussion), led to the hypothesis that variation in the number of MBEs in PAX9 could contribute to evolutionary divergence in Pax9 expression. In order to test this hypothesis, we developed a fluorescence reporter assay (see Materials & Methods). Results of Kruskal-Wallis testing based on four independent transfections derived a non-significant test statistic (H = 9.08, P = 0.06). However, additional comparisons revealed that statistical significance was heavily influenced by the removal of either cell line containing three MBEs (removing 3 MBEs cell line resulted in H = 8.45, P = 0.04, and removing 3b MBEs cell lines resulted in H = 9.41, P = 0.02). Comparison of expression across cell lines indicated that level of expression of reporter protein corresponded to number of MBEs in the 3′ UTR of reporter protein (Figure 4c). Specifically, reporter expression under the control of two MBEs was the lowest; while reporter expression under the control of four MBEs was the highest. The cell lines with two different spatial combinations of three MBEs were intermediate.

In order to determine if the differences between UTRs from different species was due to Musashi activity, we knocked down endogenous Musashi expression using RNAi and repeated the assays. RNAi knock-down of Musashi completely abrogated reporter expression for all experimental lines (H = 13.17, P<0.001), while having no effect on reporter controls possessing only SV40 3′ UTRs containing no MBEs (H = 0.05, P = 0.83; Figure 4d).

## Discussion

### Open-reading Frame Sequence Evolution

Understanding the functional significance of changes observed among sequence alignments are, as a rule, challenging. Determining the functional modification of substitutions observed in genes that are highly conserved over long evolutionary timescales, such as PAX9, is a special case of this difficulty. Within open-reading frames such genes are typified by continual purifying selection and emergence of rare variants that are often lineage specific. In this study, the PAX9 open-reading frame was found to adhere to this pattern of variation in which most genera have developed diagnostic amino acid sequences, yet a relatively small fraction of total residues were observed to vary across the broad mammalian evolutionary timescales considered. This overall pattern of variation was driven by recurrent substitution at a handful of sites, and invariance at many others. Understanding the selective basis for observed convergences is confounded by the fact that the signatures left by selection for functional change and those left by selection for functional redundancy are often the same. In genes that exhibit this type of selective history, many tests of positive selection are not empowered to detect selection. Because of this, instances of convergent sequence evolution between independent lineages convergent for phenotypic characters can be extremely informative and are often pursued by the evo-devo community [43]–[45]. Such convergences at the sequence and phenotypic levels are used as an indicator for convergent directional selection for protein function modifications. Subsequently in this section, we discuss general aspects of PAX9 open-reading frame evolution, specific instances of convergence, and the experimental possibilities for understanding observed variation.

Although PAX9 open-reading frame comparisons have previously been limited to comparisons among a narrow range of taxa, PAX9 has been shown to be highly conserved over those evolutionary time scales [7], [23], [46]. By far, exon 2, coding for the paired-binding domain, is the most highly conserved gene region, and this same pattern held true in the broader comparisons of this study. However, downstream from this region residues do vary. All but one of the 87 estimated residue substitutions among mammals occurred downstream from the paired-binding domain even though the downstream region accounts for less than 40% of the total polypeptide length. Although this region is highly variable as compared to the paired-binding domain, especially exon 3 (considering the total number of changes compared to exon length), the fact that it would be considered highly conserved in comparison to many other genes denotes an overall history of purifying selection. Pax9 is thought to physically interact with other proteins, although the specifics of these interactions are not well understood [8]. Because it is known that the C terminus of PAX9 functions in protein-DNA interactions during transcriptional activation, the function of the more variable N terminus could be specifically directed toward protein-protein interactions. Following this line of reasoning, adaptive roles of combinations of recurrent substitutions and lineage specific substitutions would relate to modifications of protein-protein interactions.

One could expect that open-reading frame variation would be greater among morphologically diversified phyllostomids relative to the craniofacially and dentally conserved vespertilionids. No indication of an increased rate in phyllostomids was observed, and there was a strong linear relationship between pairwise distance and *t_mrca_.* In fact, total number of synonymous and nonsynonymous sites was higher in vespertilionids. Given the overall evidence for stabilizing selection of the PAX9 sequence, a more reasonable expectation would be convergences between specific taxa. Phylogenetic clustering as a result of polypeptide convergence was observed both between the independently derived nectarivorous genera *Lonchophylla* and *Musonycteris* [22], [28]–[29] as well as between the pteropodids, *Eonycteris* and *Macroglossus*, and the phyllostomid *Glossophaga* (although the later relationship could be an artifact of taxon sampling and long-branch attraction). Similarly, *Micronycteris* and *Trinycteris* were found to be identical at the amino acid level and convergent for at least Thr337Ala. These two species share a similar insectivore dentition and cranial morphology, and craniometric assessments have classified these lineages as congeneric [47]. It is hypothesized that the craniofacial characteristics observed in these genera represent retention of the ancestral morphological condition [22]. However, multiple recent genetic studies have established these lineages diverged between 27–34 mya, being separated by many intermediate nodes exhibiting independent and extensive morphological divergences [22], [28]–[29].

Summarizing the open-reading frame variation observed in this study, recurrent substitution was common, and would be expected to continue if sampling across all orders was increased to that available for Chiroptera and Primates. Specific instances of convergence within bats display patterns consistent with a functional role of open-reading frame evolution in morphological distinctiveness. Yet, it is important to note that other instances of convergence were observed with no obvious morphological connection between involved lineages. Understanding the biochemical effect of such changes would require experimental validation. However, the experimental designs to test these mutational effects, such as modifications to protein stability and protein-protein interactions, would be complicated by the effects of unknown PAX9 cellular interactions, difficulties of determining specific residues of functional modification, and effects of lineage specific mutations at other relevant loci. This scenario emphasizes the experimental difficulties of understanding the contribution of open-reading frame variation to morphological evolution, especially within evolutionarily conserved genes.

### Cis-regulatory Evolution

Very little is known about the mechanisms controlling PAX9 expression. It has been postulated that PAX9 transcriptional activation is controlled by a Hoxa3 influence, as Hoxa3 mutants exhibit down regulated PAX9 expression [48]. Similarly, expression patterns of Pax9 during development are altered following full functional loss of Satb2 [49]. A cis-regulatory element has also been identified which is capable of inducing PAX9 expression in the ventromedial portion of the medial nasal process [50]. Several lines of evidence from the comparative genetic analysis of this study supported the hypothesis of a Musashi mediated post-transcriptional regulation of Pax9. The evolutionary conservation of MBEs within this gene indicates the proposed regulatory mechanism is ancient and evolutionarily conserved. However, the possibility of this regulation is contingent upon whether co-expression of Pax9 and Musashi in fact occurs. Important to the findings of the current study, it has been found that Msi-2 was among the top 100 most highly expressed novel gene discoveries in the developing mouse molar [51]. Additional survey of that microarray data set confirmed the co-expression of Msi-2 and Pax9. Further investigation of independent microarray data generated from mouse chondrocytes and osteoblasts [52], as well as human odontoblasts [53], establish the co-expression of Msi-2 and Pax9. These data are important because they confirm co-expression in developmentally relevant cell types.

Analysis of the regulatory capabilities of PAX9 3′ sequences containing different numbers of MBEs disclosed a statistically significant and directly proportional expressional effect according to MBE number. These findings were corroborated by knockdown analysis through which silencing of Musashi proteins resulted in near complete translational repression of MBE containing cell lines, but not the controls. This observation was particularly interesting, not only because it provided strong support for the hypothesized Musashi interaction, but also because it indicated interaction of regulatory protein(s) in addition to Musashi. In a regulatory scenario only involving a Musashi protein, the expressional effect of Musashi silencing on reporter proteins in MBE containing cell lines would be expected to be similar to that observed for the controls, yet this was not the case. The attenuation of reporter protein translation in MBE containing cell lines after Musashi knockdown describes a more complicated scenario, in which a combination of unidentified proteins and Musashi are required to achieve proper post-transcriptional regulation. It is possible that additional unknown regulatory motifs within the large conserved island of the 3′ UTR play a part in this putative multi-protein regulation.

An interesting finding to emerge from this study was the variation in MBE frequency within the 3′ UTR of morphologically variable phyllostomid bats, whereas the morphologically conserved vespertilionids, with the exception of one species, were invariant. Results pose the possibility that Pax9 is differentially expressed among evolutionary lineages in proportion to level of Musashi regulation. Variation of regulatory element frequency has previously been identified to affect genes involved in *Drosophila* developmental signaling pathways [54], emphasizing the role that regulatory copy number variation can have in modifying gene expression. Heterochronic effects of the Musashi-mediated regulation of PAX9 seem particularly plausible given Musashi’s established role in maintenance of pluripotency and expression timing [50], [55]–[59], and the role of PAX9 in cellular differentiation [5]–[8]. Furthermore, post-transcriptional control of expression in general is critical to proper developmental timing [60]. Yet, comparing the number of MBEs across lineages to gross morphological differences does not indicate major effects due to MBE frequency. Although evolution of Pax9 regulation is a candidate in the process of morphological diversification, changes at loci in addition to PAX9 certainly underlie observed morphologies.

In conclusion, the experimental design of this study involved comparative genetic analyses and reporter assays based on genetic variation that has evolved under the constraints of natural selection. Reporter assays yielded valuable insights into the evolutionary history of PAX9 and has supported a hypothesis for Pax9 post-transcriptional regulation. The discoveries resulting from this study were a consequence of sampling among natural variation, and these findings would not have been readily apparent through comparisons limited to model organisms. Subsequent experiments will be developed to understand how modulating Pax9 expression levels influences downstream effectors and the timing of cellular differentiation, a spatio-temporal perspective about the expression of Musashi during embryogenesis, and the specific mechanism by which Musashi-mediated post-transcriptional regulation is achieved.

## Supporting Information

Figure S1 **Neighbor-joining phylogenetic reconstructions.** A) Previously reported evolutionary relationships among all taxa included. B) Relationships estimated from PAX9 nucleotide variation in which the established relationships among families, orders, and super-orders are largely recovered. C) Relationships estimated from among amino acid predictions. Bootstrap support values based on 1000 iterations are labeled adjacent to nodes.
(TIF)

Table S1 **Sampling of bats included in this study categorized by diet and taxon.**
(DOCX)

Table S2 **PCR primer combinations.**
(DOCX)

Table S3 **Sequencing primers used in addition to PCR primers.omy.**
(DOCX)
