# Supplementary figures and images for: Contrasting Evolutionary Dynamics of the Developmental Regulator PAX9, among Bats, with Evidence for a Novel Post-Transcriptional Regulatory Mechanism
Source: PLoS One. 2013 Feb 28;8(2):e57649. doi: 10.1371/journal.pone.0057649 (PMC3585407; doi:10.1371/journal.pone.0057649)

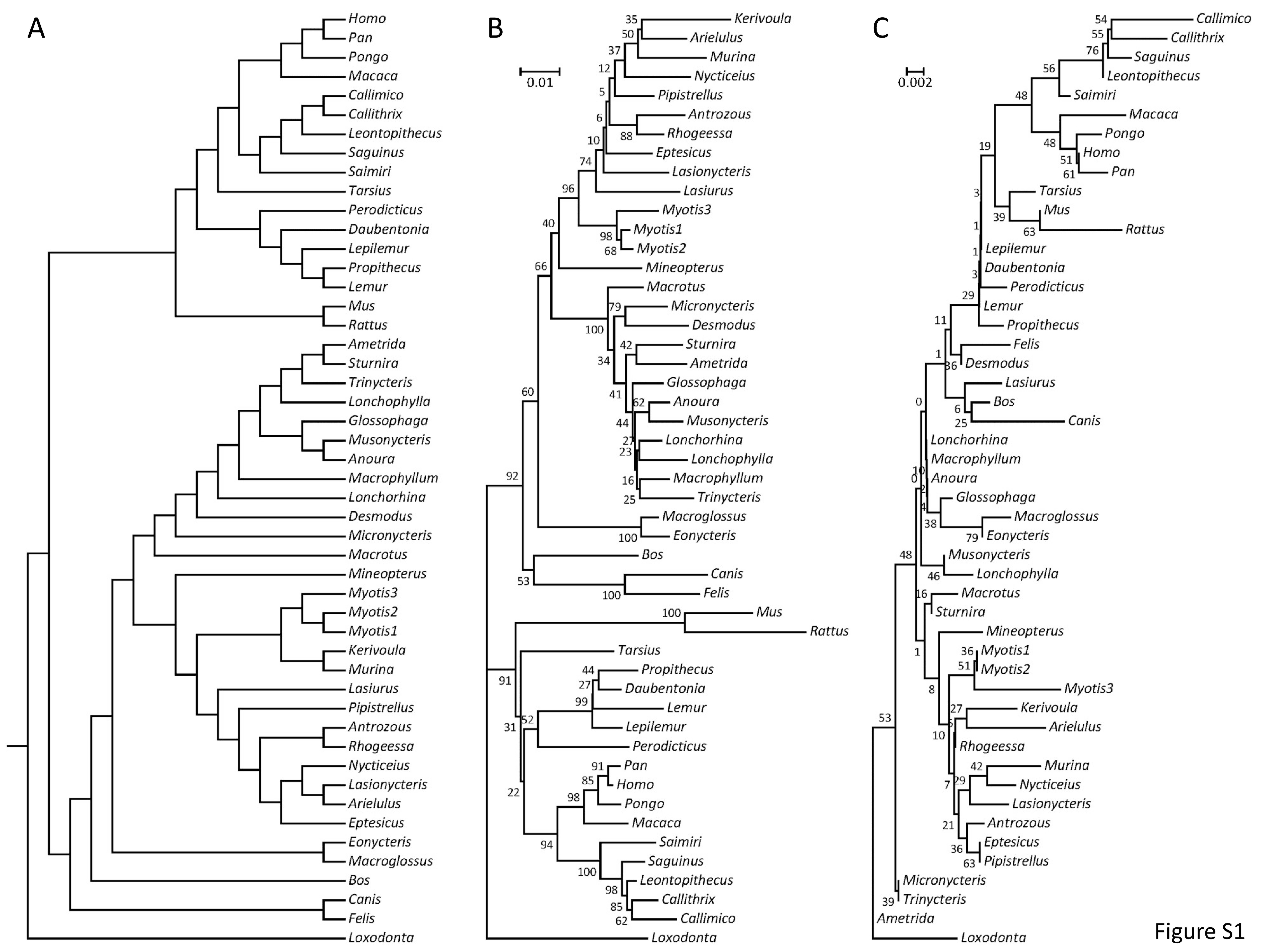

Supplement: Figure S1 — Neighbor-joining phylogenetic reconstructions. A) Previously reported evolutionary relationships among all taxa included. B) Relationships estimated from PAX9 nucleotide variation in which the established relationships among families, orders, and super-orders are largely recovered. C) Relationships estimated from among amino acid predictions. Bootstrap support values based on 1000 iterations are labeled adjacent to nodes. (TIF) [file pone.0057649.s001.tif]
