# Supplementary material for: Contrasting Evolutionary Dynamics of the Developmental Regulator PAX9, among Bats, with Evidence for a Novel Post-Transcriptional Regulatory Mechanism
Source: PLoS One. 2013 Feb 28;8(2):e57649. doi: 10.1371/journal.pone.0057649 (PMC3585407; doi:10.1371/journal.pone.0057649)
Supplement: Table S1 — Sampling of bats included in this study categorized by diet and taxon. (DOCX) [file pone.0057649.s002.docx]

Table S1. Sampling of bats included in this study categorized by diet and taxonomy.

| TK Number | Dietary Strategy | Family | Binomen |
| --- | --- | --- | --- |
| Tk101479 | Insectivore | Vespertilionidae | *Myotis elegans* |
| TK112773 | Insectivore |  | *Pipistrellus subflavus* |
| TK133305 | Insectivore |  | *Antrozous pallidus* |
| TK148823 | Insectivore |  | *Eptesicus fuscus* |
| TK148947 | Insectivore |  | *Nycticeius humeralis* |
| TK150230 | Insectivore |  | *Rhogeessa genowaysi* |
| TK152059 | Insectivore |  | *Murina suilla* |
| TK168261 | Insectivore |  | *Kerivoula hardwickii* |
| TK168346 | Insectivore |  | *Myotis horsfieldii* |
| TK168438 | Insectivore |  | *Arielulus cuprosus* |
| TK48014 | Insectivore |  | *Lasiurus cinereus* |
| TK94802 | Insectivore |  | *Lasionycteris noctivagans* |
| TK17741 | Frugivore | Phyllostomidae | *Ametrida centurio* |
| TK19119 | Insectivore |  | *Macrophyllum macrophyllum* |
| TK19556 | Nectarivore |  | *Musonycteris harrisoni* |
| TK25147 | Insectivore |  | *Micronycteris magalotis* |
| TK32036 | Insectivore |  | *Macrotus waterhousii* |
| TK101008 | Nectarivore |  | *Glossophaga soricina* |
| TK101009 | Sanguivore |  | *Desmodus rotundus* |
| TK101011 | Frugivore |  | *Sturnira ludovici* |
| TK135708 | Insectivore |  | *Trinycteris nicefori* |
| TK136045 | Nectarivore |  | *Anoura geoffroyi* |
| TK136232 | Insectivore |  | *Lonchorhina mordax* |
| TK104582 | Nectarivore |  | *Lonchophylla concava* |
| TK168342 | Insectivore | Mineopteridae | *Mineopterus schreibersii* |
| TK152068 | Nectarivore | Pteropodidae | *Macroglossus minimus* |
| TK152179 | Nectarivore |  | *Eonycteris major* |
