# Supplementary material for: Contrasting Evolutionary Dynamics of the Developmental Regulator PAX9, among Bats, with Evidence for a Novel Post-Transcriptional Regulatory Mechanism
Source: PLoS One. 2013 Feb 28;8(2):e57649. doi: 10.1371/journal.pone.0057649 (PMC3585407; doi:10.1371/journal.pone.0057649)
Supplement: Table S2 — PCR primer combinations. (DOCX) [file pone.0057649.s003.docx]

Table S2. PCR primer combinations.

| Primer Combination | Sequence | Amplifies |
| --- | --- | --- |
| P9_X1Fa/ P9_X1R | GAGACAGCGGAAGGASTTTCC **/** CCACTCACCCATTGCTCCGA | Exon 1 |
| P9_X1Fb/ P9_X2Ra | AGGATGTCNGTRACGGAGT **/** AGTTGCCGATCTTGTTACGC | Exon 1 to exon 2 |
| P9_X2Fa/P9_X2Rb | GGCTTCGCATHGTGGAACT **/**AGGATGTCNGTRACGGAGT | Exon 2 |
| P9_X2Fb/ P9_X3Rb | CATCCTGCGTAACAAGATCG **/** CGTACTTGGCBTCYTGCT | Exon 2 to exon 3 |
| P9_X3F/ P9_X3Ra | ACAGCTCCCCCTACCACAG **/** GTACTTGGCBTCYTGCTCCA | Exon 3 |
| P9_N3F/ P9_3utrRa | GGAAGGTTGACAAAGGCATATGGA **/** ARCCCTTTGAGGGGTGYAGG | Exon 4 |
| P9_X5F/ P9_3utrRc | GGACACGGVTGGCAACAT **/** TGAAACATGCTTCTGTACAACAAT | Exon 4 |
