# Supplementary material for: Contrasting Evolutionary Dynamics of the Developmental Regulator PAX9, among Bats, with Evidence for a Novel Post-Transcriptional Regulatory Mechanism
Source: PLoS One. 2013 Feb 28;8(2):e57649. doi: 10.1371/journal.pone.0057649 (PMC3585407; doi:10.1371/journal.pone.0057649)
Supplement: Table S3 — Sequencing primers used in addition to PCR primers.omy. (DOCX) [file pone.0057649.s004.docx]

Table S3. Sequencing primers used in addition to PCR primers.

| Primer | Sequence | Location | Direction |
| --- | --- | --- | --- |
| P9_X2Rc | AGTTCCACDATGCGAAGC | Exon 2 | Reverse |
| P9_N2F | AYCTGGGAGAGGCCTACT | Intron 2 | Forward |
| P9_X5Fb | CATCCACCATGGCTCCTTAY | Exon 4 | Forward |
| P9_X5R | CKGYGACAGAGTGACTACCT | Exon 4 | Reverse |
| P9_3utrF | CCCTCTAATCAAATAAGGTGACCA | 3’UTR | Forward |
| P9_3utrRb | GGCTGAGATTGCTGAACACA | 3’ UTR | Reverse |
